# 

**Published:** 1817-09

**Authors:** 


### CATALOGUE OF MEDICAL BOOKS.

Medico-Chirurgical Transactions, Vol. 8, Part I. 8vo. 10s. 6d.

A Treatise on the Nature and Cure of Gout and Rheumatism; including general Considerations on the Morbid States of the Digestive Organs, some Remarks on Regimen, and practical Observations on Gravel. By Charles Scudamore, M.D. &c. &c. The second edition, corrected and much enlarged. 8vo. 15s.

An Experimental Inquiry into the Laws of the Vital Functions; with some Observations on the Nature and Treatment of Internal Diseases. By A. P. Wilson Phillip, M.D. &c. &c. In part re-published, by Permission of the President of the Royal Society, from the Philosophical Transactions of 1815-17; with the Report of the National Institute of France on the Experiments of M. Le Gallois, and Observations on that Report. 8vo. 10s. 6d.

Principles of Diagnosis, Part II. The Diagnosis of the more general Diseases of Adults. By Marshall Hall, M.D. 8vo. 12s.

An Essay on the Nature of Light, Heat, and Electricity. By C. C. Bompass. 8vo. 7s.

### *NOTICES TO CORRESPONDENTS.*

*We were greatly obliged by the truly philosophical remarks of* Captain Bagnold, *on the supposed effect of the wind from cannon-balls. His favour arrived too late for the present Number, but shall appear in our next.*

*We have also Communications from* Dr. Kinglake, Mr. King of Mortlake, Dr. Blake, Mr. Littleton, Χειρουργος, Prophylax, Dr. Renwick of Liverpool, Vigorniensis, &c. &c.

Erratum.—In page 103, line 14, for *relative*, read *routine*.

*Our Correspondents will please to be particular in the Address of their Communications, which should be thus,*—"To the Editors of the London Medical and Physical Journal, at Mr. Souter's, No. 73, St. Paul's Church Yard; or at Mr. Adlard's, 23, Bartholomew-Close."

